# Comparing the Efficacy of IV Ibuprofen and Ketorolac in the Management of Postoperative Pain Following Arthroscopic Knee Surgery. A Randomized Double-Blind Active Comparator Pilot Study

**DOI:** 10.3389/fsurg.2018.00059

**Published:** 2018-10-03

**Authors:** Alberto A. Uribe, Fernando L. Arbona, David C. Flanigan, Christopher C. Kaeding, Marilly Palettas, Sergio D. Bergese

**Affiliations:** ^1^Department of Anesthesiology, The Ohio State University Wexner Medical Center, Columbus, OH, United States; ^2^Department of Orthopedics, Jameson Crane Sports Medicine Research Institute, The Ohio State University Wexner Medical Center, Columbus, OH, United States; ^3^Center of Biostatistics, The Ohio State University, Columbus, OH, United States; ^4^Department of Neurological Surgery, The Ohio State University Wexner Medical Center, Columbus, OH, United States

**Keywords:** ibuprofen, ketorolac, knee arthroscopy, post-operative pain management, IV non-steroidal anti-inflammatory drugs, opioids

## Abstract

**Introduction:** Acute postoperative pain following knee arthroscopy is common in orthopedic surgeries. Managing pain postoperatively combines usage of opioids and non-steroidal anti-inflammatory drugs. The aim of this clinical study was to assess the efficacy of two different analgesic treatment regimens: intravenous (IV) ibuprofen and IV ketorolac for the treatment of postoperative pain pertaining to arthroscopic knee surgery.

**Methods:** This was a single center, randomized, double-blind, parallel, active comparator clinical pilot study. Subjects were randomized to receive either IV ibuprofen, administered as two 800 mg doses or IV ketorolac, administered as a single 30 mg dose. Subjects in the ibuprofen group received 800 mg of IV ibuprofen within 2 h prior to surgery and a repeated second dose 4 h after the initial dose if they had not been discharged. Subjects in the ketorolac group received IV ketorolac 30 mg at the end of surgery, as per the manufacturer's recommendations. Pain assessments and opioid consumption data were collected up to 24 h postoperatively.

**Results:** Of 53 randomized subjects, 51 completed the study. There were 20 subjects in the ibuprofen group and 31 subjects in the ketorolac group. The median (IQR) visual analog scale (VAS) pain score at resting upon post-anesthesia care unit (PACU) arrival was 33 (12, 52) vs. 9 (2, 25) (*p* = 0.0064) for the ketorolac and ibuprofen group, respectively. The median (IQR) visual analog scale (VAS) pain score at movement upon PACU arrival was 38 (20, 61) vs. 15 (6, 31) (*p* = 0.0018) for the ketorolac and ibuprofen group, respectively. Median VAS pain scores during movement taken at subsequent 30 min intervals in the ibuprofen group were less than half that of those reported in the ketorolac group for up to 90 min after arriving in PACU. The median VAS pain scores at rest and movement in the course of 120 min−24 h after PACU arrival was not statistically significant in both groups. Rescue opioid medication during PACU stay was required in 55.0% (*N* = 11) and 83.9% (*N* = 26), with a mean amount of narcotic consumption (oral morphine conversion) of 5.53 ± 5.89 mg vs. 19.92 ± 15.63 mg for the ibuprofen and ketorolac group, respectively (*P* < 0.001). However, opioid consumption during the first 24 h after PACU discharge was not statistically significant (*p*-value = 0.637). The mean time to first rescue medication was 77.62 ± 33.03 and 55.78 ± 35.37 for the ibuprofen and ketorolac group, respectively (*p*-value = 0.0456). There were no significant differences in patient satisfaction and documented adverse events during the first 24 h.

**Conclusion:** This pilot study showed that the use of preemptive IV ibuprofen 800 mg could be considered to reduce postoperative pain and opioid consumption. Future prospective clinical trials using similar regimens should be conducted in order to gain a better understanding of how to best provide perioperative analgesic regimens.

**Clinical Trial Registration:** www.ClinicalTrials.gov, identifier NCT01650519.

## Introduction

Arthroscopic knee procedures are the most commonly performed orthopedic surgeries in the United States (1). These surgeries are mainly indicated for removal of debris, debridement of meniscal tears and cartilage flaps, recontouring of cartilage flaps, arthroscopic reconstruction of ligaments and transplantation of the meniscus, and resection of the synovial joint (1). Acute postoperative pain following knee arthroscopies is a common occurrence in orthopedic patients (2–5). Around 80% of patients undergoing a surgical procedure experience postoperative pain, with 86% of those reporting pain as moderate to severe (2–5). Managing pain after arthroscopic knee surgery frequently entails the use of a combination of opioids and non-steroidal anti-inflammatory drugs (NSAIDs) (6, 7).

NSAIDs have historically been used to decrease pain and inflammation in a variety of clinical settings (8). Furthermore, to effectively manage pain and inflammation in orthopedic and non-orthopedic surgeries, the combined use of NSAIDs and opioid analgesics has been shown to be more effective than just using them individually as a monotherapy (9–11). However, opioid administration is often associated with undesirable side effects including, but not limited to, nausea, emesis, drowsiness, moderate sedation, respiratory depression, pruritus, urinary retention, ileus, and failure to reduce pain caused by tissue inflammation (3, 4, 12–17). NSAIDs maintain persistent levels of prostaglandin inhibition by obstructing the cyclooxygenase (COX) enzyme in the peripheral nociceptors, thereby preventing the sensitization of pain receptors (2, 8). NSAIDs also act on the central nociceptor by blocking COX-2 and subsequently inhibiting the production of prostaglandin E2 in the spinal dorsal horn, thereby activating medullary and cortical regions causing central sensitization which reduces the pain threshold around unharmed tissue (8).

Ibuprofen is the most popularly consumed over-the-counter and prescribed NSAID in the world; it functions as a non-selective inhibitor of cyclo-oxygenase-1 (COX-1) and cyclooxygenase-2 (COX-2) (18, 19). Oral ibuprofen is commonly used for its analgesic, antipyretic, and anti-inflammatory properties and its higher rate of tolerability (2, 3, 20–22). The inhibition rate of COX-1 to COX 2 of ibuprofen is 2.5:1, which indicates a reduced risk of bleeding or gastrointestinal problems, its anti-inflammatory and analgesic effects are associated with the inhibition of COX-2 (6, 19). Ibuprofen has been found to provide pain control that is at least equivalent to narcotics, while it is not associated with respiratory depression or other opioid related side effects (23). In June 2009, the American Food and Drug Administration (FDA) approved IV ibuprofen for the treatment of mild to moderate pain as a single therapy and for moderate to severe pain as an adjunct to opioids (20, 21). The half-life of IV Ibuprofen is ~2–3 h and the maximum recommended dose limit is 3,200 milligrams (mg) (14, 21). A postoperative pain study demonstrated that 800 mg of IV ibuprofen every 6 h is well tolerated, resulting in fewer side effects, reduction in pain, and decreased morphine consumption when compared to placebo (2, 3, 20, 24).

In contrast, Ketorolac is an FDA approved antipyretic and analgesic IV NSAID that has been used for the indication of postoperative pain (25). Before 2009, ketorolac was the only IV NSAID approved to treat pain by the FDA in the United States; however, its pre-operative use is contraindicated according to the manufacturer's dosing instructions (26). Multiple fatalities have been reported secondary to ketorolac's gastrointestinal and operative site bleeding side effects (27). Ketorolac has been shown to increase the incidence of intraoperative blood loss and postoperative bleeding when it is administered before or during surgery (25, 28, 29). It has a ratio of COX-1 to COX-2 inhibition of 330:1, which explains the high risk of side effects and why its use is controversial in most perioperative settings (6). Due to its restriction to postoperative administration, ketorolac is more commonly used for the treatment of acute postoperative pain (4). The IV bioavailability of ketorolac is similar to its oral bioavailability, which is about 80–100%, with a peak plasma concentrations of 30–60 min after administration, resulting in rapid analgesia and a half-life of 5–6 h approximately (30, 31).

Postoperative pain management is one of the principal concerns of surgeons and anesthesiologists (2, 12). The literature shows sufficient data supporting the efficacy of a multimodal analgesic regimen for postoperative pain (3, 12). One of the main reasons for insufficient pain control is the patient's and surgeon's reticence to use opioid agents due to their undesirable side effects that could lead to delay of discharge time or subsequent addiction; hence, the use of a multimodal pain regimen could be beneficial and is widely supported (2). Studies have shown that a multimodal approach of postoperative pain management has been useful in controlling pain and reducing opioid consumption with its associated adverse effects (17).

Therefore, a prospective, two-armed, randomized, double-blind pilot study was created to assess the feasibility of the study design, and the efficacy of IV ibuprofen compared to IV ketorolac for the treatment of postoperative pain as measured by patient pain intensity with a visual analog scale (VAS) in patients undergoing arthroscopic knee surgery at The Ohio State Wexner Medical Center. The decision of administrating two different regimens with pre-emptive analgesia (IV ibuprofen) and postoperative analgesia (IV ketorolac) in the study was made considering the restriction of pre-operative administration of ketorolac and pharmacokinetic profile of both IMPs (4). We hypothesized that the administration of IV ibuprofen prior to surgery was a more effective and opioid-sparing analgesia regimen than the administration of IV ketorolac at the end of surgery on subjects undergoing arthroscopic knee surgery.

## Methods

This was a single center, randomized, double-blind, parallel, active comparator clinical pilot study designed to assess the efficacy, and safety of IV ibuprofen (Caldolor™, Cumberland Pharmaceuticals, Nashville, TN) for pain control following arthroscopic knee surgery. This study was approved by the Western Institutional Review Board (WIRB approval No. 20120992). The clinical trial registry of this study is NCT01650519.

Subjects scheduled to undergo arthroscopic knee surgery under general anesthesia were enrolled in this study. Eligible subjects, 18 years and older, provided a written informed consent and self-reported their pain level by use of a paper Visual Analog Scale (VAS). Subjects with inadequate IV access, a history of allergy or hypersensitivity to any component of ibuprofen or other NSAIDs, aspirin (or aspirin related products), opioids or COX-2 inhibitors, or had used analgesics <8 h prior to surgery were excluded from the study. Subjects with active significant anemia, history of asthma or heart failure, and recent history of chronic NSAIDs or opioid use were also excluded from the study. Women who were pregnant were not enrolled in the study, and epidural anesthesia and nerve blocks were prohibited.

The randomization method was simple randomization: an un-blinded research pharmacist used an online random list generator (http://randomization.com/) that was created prior enrollment period (32). Subjects who met all the inclusion and none of the exclusion criteria during screening/baseline were randomized in a 1:1 ratio to receive either two doses of 800 mg IV ibuprofen or a single dose of 30 mg ketorolac (15 mg for subjects >65 years of age).

A formal power analysis for sample size calculation was not performed for this trial since it was considered a pilot study. Therefore, the study intended to enroll 50 complete subjects (25 in each group) in order to provide us with descriptive safety and efficacy data for analysis. All data analyses were performed using SAS 9.4 (SAS Institute Inc., Cary, NC). Descriptive statistics were used to describe patient characteristics including means and standard deviations or medians and interquartile range for continuous variables and frequencies and proportions for categorical variables. The Shapiro-Wilk test was used to test for normality of continuous variables. Continuous variables were compared between groups using standardized *t*-test or Wilcoxon Rank sum test, where appropriate, and categorical variables were compared using Chisquare or Fisher's Exact tests.

Investigational medicinal product (IMP) was administered at three time points: within 2 h before surgery (study hour 0), end of arthroscopy procedure, and 4 h after the first dose of IMP (hour 4). In order to maintain the blinding process throughout the study, pharmacists were the only un-blinded research personnel in charge of preparing and dispensing IMP in prefilled bags/injections with the subjects' information and IMP information as follows: 800 mg of IV ibuprofen/placebo (normal saline, sodium chloride 0.9%) and 30 mg of ketorolac/placebo; the syringe used for the ketorolac/placebo injection was wrapped with tape in order to conceal any disparity in the visual presentation of the solutions. The ibuprofen group received 800 mg IV ibuprofen at hour 0 and hour 4 and matching placebo injection at the end of surgery. The ketorolac group received matching placebo at hour 0 and 4 and 30 mg of IV ketorolac at the end of surgery. The second dose of ibuprofen was administered only if the subject remained in the hospital at hour 4. All subjects remained blinded throughout the study and were informed during the consenting process of the possibility of receiving an additional dose of ibuprofen/placebo.

Perioperative medication, as well as the anesthesia regimen, was standardized in both groups. Subjects received IV midazolam 0.03–0.09 mg/kg as pre-induction medication. Subjects underwent induction of anesthesia with IV lidocaine 1 mg/kg and propofol 2 mg/kg, and anesthesia was maintained with sevoflurane in air/oxygen. Analgesia during surgery was provided with fentanyl 1.0 mcg/kg. In addition, IV 4–8 mg dexamethasone and 4 mg ondansetron were given for postoperative nausea and vomiting prophylaxis.

The primary aim of this clinical pilot study was to assess the efficacy of IV ibuprofen compared to IV ketorolac for the treatment of postoperative pain in subjects undergoing arthroscopic knee surgery. Therefore, pain at rest and during movement was assessed by asking the patient to rate their pain level by using a visual analog scale (VAS) was collected every 30 min in post anesthesia care unit (PACU) until discharge, and at 24 h after end of surgery. PACU rescue analgesic was managed with IV hydromorphone 0.5 mg as needed. Subjects were discharged with a prescription of 800 mg oral ibuprofen, every 6 h as needed and oxycodone/acetaminophen (5/325 mg), every 4 h as needed. Subjects were instructed to complete a diary to record VAS pain at 24 h and the amount of rescue pain medication taken after discharge. In addition to demographic data, adverse event reports during the first 24 h, the time that rescue medication was first used and the total amount taken in the first 24 h were also recorded as secondary outcomes.

In addition, subjects received a patient satisfaction questionnaire to be completed and sent by email at 24 h after surgery.

## Results

**Figure 1:**
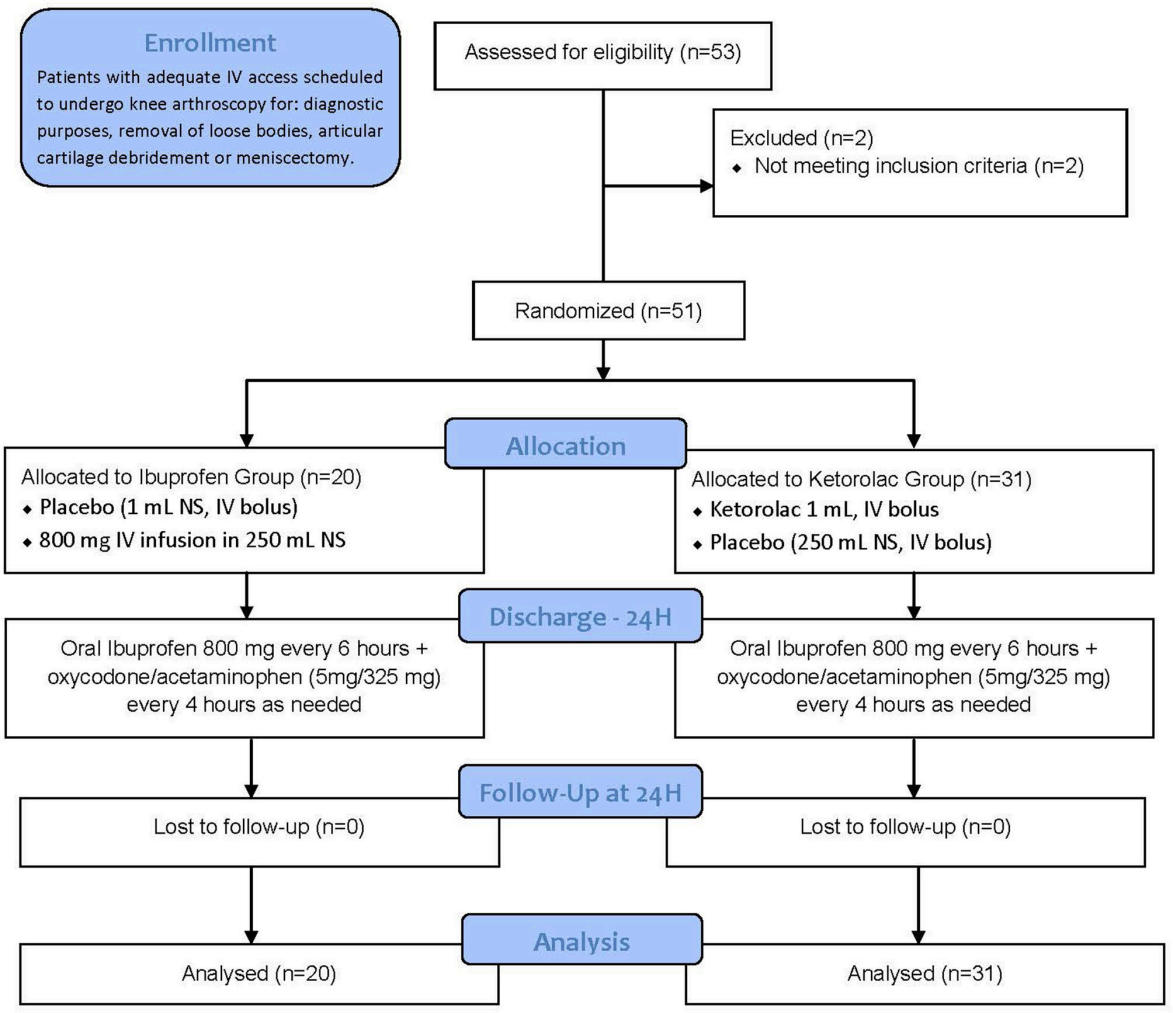
Patient screening flow diagram. IV = intravenous; NS = normal saline; mL = milliliters.

A total of 53 subjects were enrolled at The Ohio State University Wexner Medical Center between September 2012 and December 2012; however, two of them were considered screen failures for failing to meet all inclusion criteria or for meeting any of the exclusion criteria, and were subsequently excluded from this study. After the un-blinding process, we learned that 20 subjects were allocated to the ibuprofen group and 31 subjects to the ketorolac group due to a human error made by the un-blinded research pharmacist. As a result, 51 subjects completed the study, but only 8 (40.0%) and 16 (51.6%) subjects from the ibuprofen and ketorolac group respectively received the second dose of IMP (placebo/ibuprofen 800 g) due to limited PACU stay length. The patient screening flow diagram is displayed in Figure 1.

Table 1 shows the demographics and surgical variables for the study population that received at least one dose of IMP. There were no statistically significant differences between the drug groups in demographics, length of surgery, or length of PACU stay.

**Table 1:** Demographics and surgical variables.

**Characteristic**	**Overall (*n* = 51)**	**Ibuprofen (*n* = 20)**	**Ketorolac (*n* = 31)**	***P*-Value**
Age, mean (SD), years	43.7 ± 12.7	42.32 ± 12.37	44.6 ± 13.03	0.5455
Male, *n* (%)	35 (68.6%)	14 (70%)	21 (67.74%)	0.8653
Female, *n* (%)	16 (31.4%)	6 (30%)	10 (32.26%)	0.8653
Race				0.2355
Black, *n* (%)	4 (7.84%)	3 (15%)	1 (3.23%)	
Caucasian, *n* (%)	46 (90.20%)	17 (85%)	29 (93.55%)	
Hispanic, *n* (%)	1 (1.96%)	0 (0%)	1 (3.23%)	
Weight, mean (SD), kg	90 ± 21.85	89.7 ± 21.2	90.2 ± 22.61	0.9368
Length of Surgery, mean (SD), min	26.18 ± 21.79	27.25 ± 14.48	25.48 ± 25.64	0.7551
Length of PACU, mean (SD), min	108.59 ± 28.55	109.35 ± 31.32	108.10 ± 27.13	0.8802

*SD, standard deviation; n, total number; %, percentage; kg, kilogram*.

**Figure 2:**
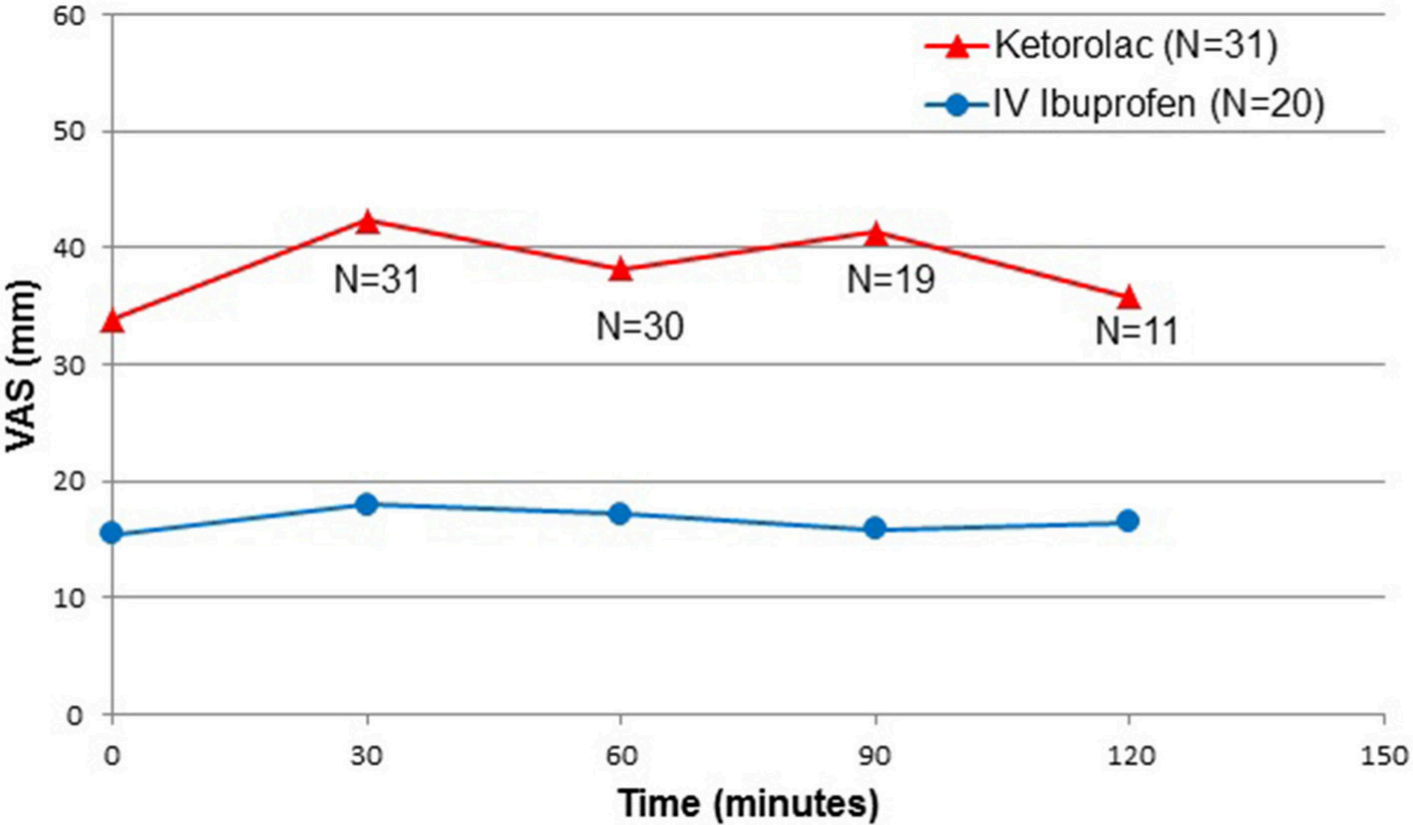
Postoperative pain intensity, at rest. N = number; VAS = visual analog scale; Time 0 = Post-anesthesia Care Unit arrival time.

Immediately upon arriving in PACU following surgery, the median (IQR) resting VAS pain score was 33 (12, 52) millimeters (mm) for the ketorolac group and 9 (2, 25) mm for the ibuprofen group (*p* = 0.0064). Subsequent VAS pain scores at rest of the operative joint demonstrated more than a two-fold reduction in the ibuprofen group (Figure 2).

**Figure 3:**
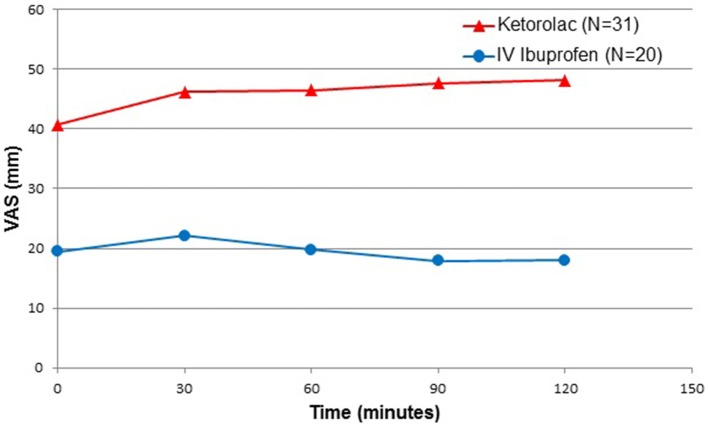
Postoperative pain intensity, at movement. N = number; VAS = visual analog scale; Time 0 = Post-anesthesia Care Unit arrival time.

The median (IQR) VAS pain scores during movement upon PACU arrival was 38 (20, 61) for the ketorolac group and 15 (6, 31) mm for the ibuprofen group (*p* = 0.0018). Median VAS pain scores during movement taken at subsequent 30 min intervals in the ibuprofen group were less than half that of those reported in the ketorolac group for up to 90 min after arriving in PACU (Figure 3).

The median VAS pain scores at rest and movement in the course of 120 min−24 h after PACU arrival did not differ with statistical significance between both groups.

A total of 37 (72.5%) subjects required rescue opioid medication during PACU stay and prior to discharge. Approximately 55.0% (*N* = 11) of the subjects in the ibuprofen group required postoperative rescue pain opioid medication during PACU stay, as compared to 83.9% (*N* = 26) in the ketorolac group (*p* = 0.0241).

**Figure 4:**
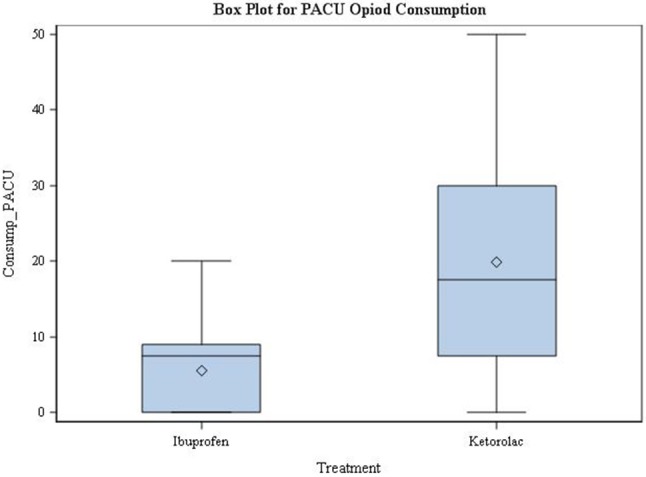
Post-Anesthesia Care Unit Opioid Consumption. PACU = Post-Anesthesia Care Unit; Consump = consumption; Time 0 = Post-anesthesia Care Unit arrival time.

Up until the time of PACU discharge, the mean amounts of narcotic consumption (oral morphine conversion) in the ibuprofen group was significantly lower than in the ketorolac group: 5.53 ± 5.89 mg, vs. 19.92 ± 15.63 mg, respectively (*P* < 0.001) (Figure 4).

During the first 24 h following PACU discharge, the mean total of narcotic medication consumption was 12.41 ± 16.56 mg in the ibuprofen group and 11.25 ± 15.91 mg in the ketorolac group. This was not statistically significant (*p*-value = 0.637).

**Figure 5:**
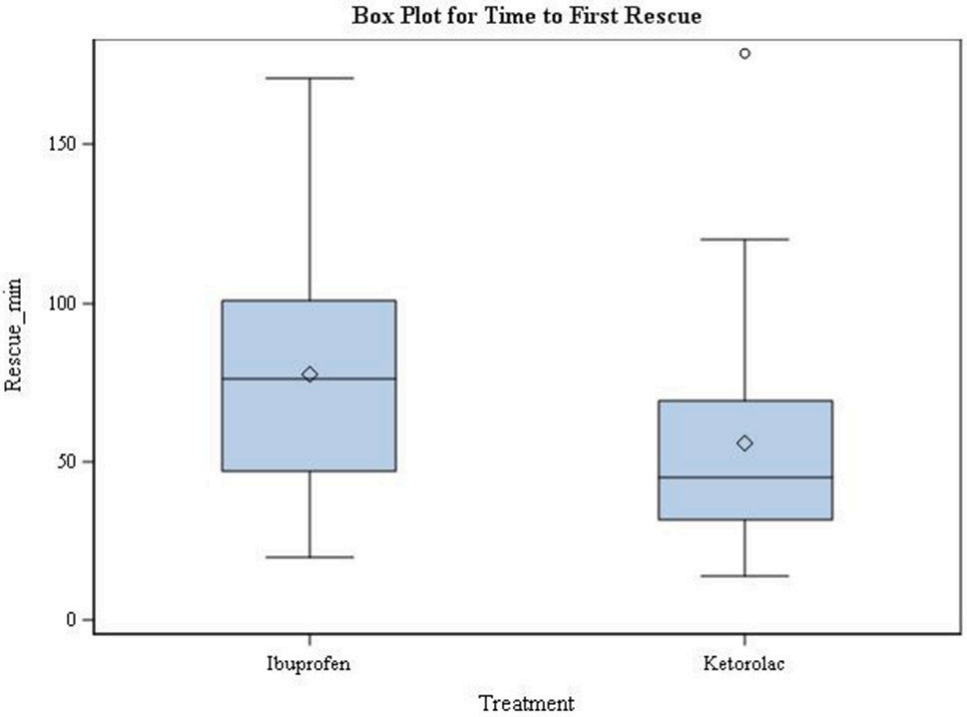
Time of first postoperative narcotic medication used. min = minutes; Time 0 = Post-anesthesia Care Unit arrival time.

The mean time to first rescue medication was 77.62 ± 33.03 and 55.78 ± 35.37 for the ibuprofen and ketorolac group respectively, there was a statically significant difference between both groups (*p*-value = 0.0456) (Figure 5).

There were no significant differences in patient satisfaction and documented adverse events during the first 24 h between both groups.

## Discussion

The primary aim of this clinical pilot study was to assess the efficacy of IV ibuprofen compared to IV ketorolac for the treatment of postoperative pain in subjects undergoing arthroscopic knee surgery. The results of this study demonstrated that the use of IV ibuprofen, compared to IV ketorolac, significantly lowered postoperative pain scores and opioid consumption in subjects undergoing arthroscopic knee surgeries.

Current literature suggests that IV ibuprofen be ideally used on a short-term basis, such as in inpatient and outpatient surgical procedures, as few safety concerns on its short-term use have been documented (2, 3, 12, 13).

The ibuprofen drug packet insert (Caldolor™, Cumberland Pharmaceuticals, Nashville, TN) recommends infusing IV ibuprofen over a time span of no less than 30 min, but studies have shown that rapidly infusing over 5–7 min is safe and leads to higher maximum plasma concentration in a shorter amount of time (24). Similarly, Bergese et al. conducted a multi-center, open label study to assess the safety profile and efficacy of IV ibuprofen (33). In this study, 150 patients, who were hospitalized for pain or fever, received IV ibuprofen over 5–10 min (33). Bergese reported a 52% average decrease in patient reported VAS compared to their baseline pain level (33). The study demonstrated that the most common adverse events of IV ibuprofen administration was pain at infusion site, nausea, flatulence, anemia, and bradycardia (33).

A 2010 prospective, double-blind, placebo-controlled trial was conducted by Singla et al. involving 185 patients undergoing elective orthopedic surgeries consisting mainly of knee and hip replacements (12). The first dose of ibuprofen was administered pre-operatively, similar to the aforementioned study and their results are consistent with our findings in terms of reduction in postoperative pain scores and opioid consumption (12). Patients were treated with either 800 mg IV ibuprofen or placebo every 6 h for at least 24 h (12). The results showed reduction in both pain and opioid consumption in the ibuprofen group compared to placebo group during the 6–28 h postoperative period (12).

Kroll et al. conducted a study involving 319 females undergoing abdominal hysterectomy, they were randomized into either a group receiving 800 mg IV ibuprofen with morphine or a group receiving placebo with morphine for postoperative pain control (3). The study showed that the patients who received ibuprofen had a 19.5% reduction in morphine use and reported 21% lower pain scores in the first 24 h, compared to ones in the placebo group (3). This study differs from our study in that they administered the first dose of ibuprofen/placebo a few minutes before the end of surgery and patients received a dose every 6 h for at least 2 days; whereas, we administered the first dose of ibuprofen within 2 h before surgery and a second dose 4 h later, if they were still in the PACU (3).

Southworth et al. conducted a study which involved 406 patients who underwent a wide variety of elective orthopedic and abdominal surgeries. The patients were randomized using a 1:1:1 scheme and were assigned to receive either 400 mg IV ibuprofen, 800 mg IV ibuprofen, or placebo at the initiation of wound closure, and then every 6 h for at least the first 48 h (2). The study reported that only the 800 mg IV dose level of ibuprofen improved postoperative pain at rest and during movement compared to placebo throughout the first 24 h after surgery. 400 mg IV ibuprofen only provided significant analgesia at rest and during movement compared to placebo during measures taken 6–24 h after surgery, yet both ibuprofen dosages significantly reduced opioid consumption (2). The incidence of adverse events had similar prevalence among both ibuprofen dosage groups, but dizziness was observed in patients in the 800 mg dose group (*P* = 0.011) (2). The study design differs from ours in several aspects. The subjects from this study received IV ibuprofen every 6 h for at least 2 days and the initial dosage was administered a few minutes before the end of surgery. In addition, their study protocol allowed the use of perioperative IV morphine, but not 45 min prior to wound closure (2).

A few studies compared the administration of preoperative vs. postoperative IV ketorolac in regards to their efficacy on pain control and occurrence of known side effects.

Fragen et al. performed a placebo-controlled study wherein the patients received 4 doses within 24 h of either 30 mg IV ketorolac or placebo about an hour before the end of total knee arthroplasty surgery (28). They found a 27% decrease in the use of morphine in the patients who received intraoperative ketorolac and a 6% increase in the incidence of blood loss in ketorolac group compared to placebo (28). This study differs from our study in ketorolac administration time. This study dosed ketorolac 45 min before surgery; and ours dosed at the end of surgery (28).

A placebo-controlled study by Fletcher et al. compared the effect of preoperative and postoperative IV ketorolac administration on pain level and postoperative morphine consumption in patients undergoing total hip replacement (34). Twenty patients received 60 mg IV ketorolac before surgery and 2 mL saline after surgery (PRE); 20 patients received 2 mL saline before surgery and 60 mg IV ketorolac immediately after surgery (POST); 20 patients received 2 mL saline before and after surgery (control). The researchers reported no significant reduction in the patient's pain scores in either of the pre- and postoperative ketorolac groups (34). Additionally, no significant difference was seen in terms of opioid consumption among the patients that received ketorolac after surgery and the control group (34). However, there was a significant difference among the patients that received ketorolac before surgery and the other two groups, but only during the immediate postoperative period (34). Furthermore, there were two patients in each non-control group that suffered from postoperative blood loss (34). There was no significant difference in the number of transfusions required within the 3 groups (34). Some of the limitations of this study included the small sample size of 20 patients in each of the 3 groups, which would not accurately represent the side effect profile of ketorolac (34).

A 1997 clinical trial conducted by Balestrieri et al. explored the effect of IV ketorolac administered intraoperatively vs. postoperatively on pain scores and opioid consumption in 248 patients undergoing elective hysterectomy or myomectomy surgery. Patients were randomly assigned into three groups to receive ketorolac/placebo on a dosing schedule of dose 1 given one-half hour before the expected end of surgery, dose 2 given upon awakening in the PACU, and doses 3, 4, and 5 given at 6, 12, and 18 h, respectively, after dose 2. Group 1 patients received placebo (saline) for dose 1, ketorolac 60 mg for dose 2, and ketorolac 30 mg for doses 3, 4, and 5. Group 2 patients received ketorolac 60 mg for dose 1, placebo for dose 2, and ketorolac 30 mg for doses 3, 4, and 5. Group 3 patients received placebo for all doses. The results showed that average VAS scores before dose 2 were significantly lower in the intraoperative ketorolac group (Group 2) than the postoperative ketorolac group (Group 1) during the immediate postoperative period. Group 2 patients also had decreased morphine consumption as compared to placebo. Additionally, both ketorolac groups (Group 1 and Group 2) had significantly higher values for ease of nursing care and tolerability as compared to placebo (Group 3). It is important to note that intraoperative ketorolac only improved early postoperative pain scores, and both intraoperative and postoperative ketorolac resulted in improved overall pain scores at similar intensities compared to the placebo group. Thus, due to the lack of clinically significant differences in analgesia between the two ketorolac dosing regimens, the researchers suggest that clinicians weigh the risks vs. benefits involved in perioperative ketorolac administration (35).

Chow et al. conducted a double-blind prospective study in 2001 to evaluate the efficacy of preemptive ketorolac administration for pain control and opioid consumption after laparoscopic urologic surgery. Fifty-five patients completed the study, having been randomly assigned to receive 15–30 mg IV ketorolac or matching placebo injection prior to surgery. The average pain score was 2.2 and 4.5 for the ketorolac and placebo groups, respectively (*P* < 0.005). The average volumes of total morphine used by each group after surgery were 39.2 mg (ketorolac) and 62.5 mg (placebo) (*P* = 0.077). The researchers found no adverse effects associated with pre-operative ketorolac administration; there was no change in creatinine levels between either group, and there were no instances of bleeding diathesis or gastrointestinal hemorrhage (36).

In light of the limited incidences of adverse events typically associated with preoperative/intraoperative ketorolac administration, it may be safe for future studies to replicate our protocol with preoperative IV ibuprofen and preoperative IV ketorolac administration for postoperative analgesia. Such a study would remove the confounder that our study encountered, namely the pre-emptive vs. postoperative of an NSAID for pain control.

Two studies have compared ibuprofen with ketorolac for the treatment of postoperative pain.

In a prospective double-blinded randomized trial, Dr. Charles Mixter III compared the analgesic effect of 800 mg oral ibuprofen an hour before surgery and 60 mg IV ketorolac at the time of trocar insertion in 70 patients undergoing laparoscopic hernia repair. Postoperative pain was assessed 18 and 24 h after discharge from the hospital, and it was discovered that the analgesic effects of the two treatments did not differ significantly at time of discharge and 18 h after discharge. Mixter concluded that preoperative ibuprofen is superior for postoperative analgesia compared to intraoperative ketorolac due to ibuprofen's low cost and the reduced risk for adverse reactions to the drug (37). This study differs from ours in three aspects. Firstly, we used preemptive intravenous ibuprofen, while Mixter et al. used preemptive oral ibuprofen, which undergoes first pass metabolism before entering circulation. Secondly, we administered IV ketorolac after the completion of surgery, as instructed by ketorolac's drug label. In contrast, Mixter administered IV ketorolac intraoperatively. Thirdly, we asked subjects to rate their postoperative pain during the immediate postoperative recovery when pain is most severe; Mixter evaluated postoperative pain at discharge from recovery.

A 1994 study of 50 patients undergoing laparoscopic tubal ligation by Higgens et al. failed to find a significant difference between placebo and 800 mg oral ibuprofen or 60 mg IV ketorolac in the reduction of postoperative pain or side effects when administered preoperatively. Eighty percent of subjects in the control group, and in and 73% of subjects in either treatment group required parenteral morphine while in surgical recovery. The researchers proposed the nature of the surgery, which is unique in that it results in additional fallopian tube injury, as the reason why their results differed from the many other studies which have confirmed the analgesic effects of both ibuprofen and ketorolac. The additional prostaglandin-mediated pain may be worse than other laparoscopic procedures, hence rendering NSAID administration at their established doses relatively ineffective in managing pain (38).

Our study had a few limitations to be considered. The first limitation was the design of the study; this was a pilot study with 51 patients without a control group or a power analysis. Despite this limitation, there was an allocation error by unblinded research personnel (pharmacy); subjects were intended to be randomized at a 1:1 ratio. However, upon unblinding at the conclusion of the study, it was found that patients were distributed at nearly 3:2 ratio between the ketorolac and ibuprofen groups. Another limitation was that the design of the study was made considering the label administration instructions and reported half-life of both investigation products. Therefore, IV ketorolac/placebo was administered only one time at the end of surgery, in contrast to the IV ibuprofen/placebo administration, where the first dose was given within 2 h prior to surgery and the second dose was given 4 h after the first dose (only on subjects that were not discharged from PACU at Hour 4). Consequently, 24 subjects (8 and 16 in the ibuprofen and ketorolac groups, respectively) received the second dose of IV ibuprofen/placebo. Furthermore, the varied administration of the two drugs may have introduced a confounder to our study due to the fact that pre-emptive systemic ibuprofen was already present in plasma levels at the time of surgical trauma. On the other hand, the half-life of IV ibuprofen and IV ketorolac is ~2–3 h and 5–6 h, respectively (14, 21, 30, 31). Therefore, these facts could have interfered with postoperative pain and opioid consumption levels assessments.

## Conclusion

This pilot study showed that the use of preemptive IV ibuprofen 800 mg could be considered to reduce postoperative pain and opioid consumption within the first 24 h after arthroscopy surgery. In conclusion, this study showed that a multimodal analgesia regimen including the use of IV ibuprofen administration could be an adequate regimen to reduce postoperative opioid consumption and pain. Future prospective clinical trials using similar regimens should be conducted in order to gain better understanding of how to best provide multimodal perioperative analgesic regimens.

## Author's note

The abstract of this paper was presented at the 2013 American Society of Anesthesiologists Conference as a poster presentation with interim findings. The poster's abstract was published on 2013 The Anesthesiology Annual Meeting website (39).

## Author contributions

AU, FA, and SB contributed with the conception and design of the study. AU, FA, and SB organized the database and wrote the first draft of the manuscript. MP performed the statistical analysis. DF, CK, and MP wrote sections of the manuscript. All authors contributed to manuscript revision, read and approved the submitted version.

### Conflict of interest statement

The authors declare that the research was conducted in the absence of any commercial or financial relationships that could be construed as a potential conflict of interest.
